# Automatic categorization of diverse experimental information in the bioscience literature

**DOI:** 10.1186/1471-2105-13-16

**Published:** 2012-01-26

**Authors:** Ruihua Fang, Gary Schindelman, Kimberly Van Auken, Jolene Fernandes, Wen Chen, Xiaodong Wang, Paul Davis, Mary Ann Tuli, Steven J Marygold, Gillian Millburn, Beverley Matthews, Haiyan Zhang, Nick Brown, William M Gelbart, Paul W Sternberg

**Affiliations:** 1 Howard Hughes Medical Institute and Biology Division, California Institute of Technology, Pasadena, CA 91125, USA; 2 Wellcome Trust Sanger Institute, Wellcome Trust Genome Campus, Cambridge, CB10 1SA, UK; 3 Department of Genetics, University of Cambridge, Downing Street, Cambridge, CB2 3EH, UK; 4 Department of Molecular and Cellular Biology, Harvard University, Cambridge, MA 02138, USA; 5 The Gurdon Institute and Department of Physiology, Development & Neuroscience, University of Cambridge, Tennis Court Road, Cambridge, CB2 1QN, UK

## Abstract

**Background:**

Curation of information from bioscience literature into biological knowledge databases is a crucial way of capturing experimental information in a computable form. During the biocuration process, a critical first step is to identify from all published literature the papers that contain results for a specific data type the curator is interested in annotating. This step normally requires curators to manually examine many papers to ascertain which few contain information of interest and thus, is usually time consuming. We developed an automatic method for identifying papers containing these curation data types among a large pool of published scientific papers based on the machine learning method Support Vector Machine (SVM). This classification system is completely automatic and can be readily applied to diverse experimental data types. It has been in use in production for automatic categorization of 10 different experimental datatypes in the biocuration process at WormBase for the past two years and it is in the process of being adopted in the biocuration process at FlyBase and the *Saccharomyces *Genome Database (SGD). We anticipate that this method can be readily adopted by various databases in the biocuration community and thereby greatly reducing time spent on an otherwise laborious and demanding task. We also developed a simple, readily automated procedure to utilize training papers of similar data types from different bodies of literature such as *C. elegans *and *D. melanogaster *to identify papers with any of these data types for a single database. This approach has great significance because for some data types, especially those of low occurrence, a single corpus often does not have enough training papers to achieve satisfactory performance.

**Results:**

We successfully tested the method on ten data types from WormBase, fifteen data types from FlyBase and three data types from Mouse Genomics Informatics (MGI). It is being used in the curation work flow at WormBase for automatic association of newly published papers with ten data types including RNAi, antibody, phenotype, gene regulation, mutant allele sequence, gene expression, gene product interaction, overexpression phenotype, gene interaction, and gene structure correction.

**Conclusions:**

Our methods are applicable to a variety of data types with training set containing several hundreds to a few thousand documents. It is completely automatic and, thus can be readily incorporated to different workflow at different literature-based databases. We believe that the work presented here can contribute greatly to the tremendous task of automating the important yet labor-intensive biocuration effort.

## Background

The phenomenal growth in bioscience literature has posed a great challenge in information retrieval both for general researchers and those whose task it is to extract such information from the literature (biocuration) [1,2]. Text mining for bioscience data is an active research area and many tools are emerging [3-5].

Extensive work has been done on the categorization of papers with experimental information and the extraction or and retrieval of content from the text in biomedical literature. The most extensively studied data types involve protein-protein interaction [6,7]. Categorization of other data types such as tumor, allele, gene expression and Gene Ontology (GO) terms, and so forth, have been reported [8,9]. Efforts to address different information needs of diverse users in the biomedical field have been made by using a multi-dimensional categorization and annotation scheme through identifying contents and classifying papers rich with multiple categories with sufficient generality and applicability to diverse subject areas [10-13].

Although some applications are starting to be incorporated into the biocuration workflow at some databases [14,15], biocuration remains largely a manual effort. Since 2002, text classification has been listed as one of the tasks in several grand challenges [3,4,16], and many machine-learning methods have been developed for this task. Attempts have been made to automate text classification but the performance is not yet satisfactory for a fully automated curation process [6].

Various machine-learning methods have been successfully applied to text categorization, including naïve Bayesian learning [17], neural networks [18], instance-based learning [19], maximum entropy [20], and Support Vector Machines (SVM) [21]. SVM was first successfully applied to text classification in 1991 [21] and has been shown to perform better than other machine learning methods in some cases, especially when there are few training data [22,23]. Briefly, for a given data type, i.e., class, SVM learns a binary classifier from its positive and negative training documents presented as data vectors, by formulating and solving a quadratic optimization problem. The classifier is defined by a hyperplane with a maximum margin that separates the sample space to a positive and a negative half space containing positive and negative sample points respectively (Additional File 1, Figure S1). The process of applying SVM to text classification includes the following steps: selection of features (words) to represent the documents; construction of the training data vector where the elements of the vector are scores derived from the feature usage in the documents, using a certain term (feature) weighting scheme; learning a classifier by supplying the training data vector into an SVM library with the chosen SVM kernel function and parameters; and finally, classifying a new document by converting it to a data vector and feeding it into the classifier.

Both feature selection and term weighting schemes are active research areas and various methods have been developed [24-30]. The SVM algorithm, originated by Vapnik [31], has been implemented in several libraries, such as SVM-light [32] and LIBSVM [33], each with a number of selections of kernel functions. However, it is often not clear at the outset what method is most suitable for each of the steps described above when applying the SVM algorithm to a particular classification problem [33], and experiments with each of these areas usually need to be conducted to find or develop the most suitable method for each.

We describe here the successful application of an SVM procedure for the identification of ten, fifteen, and three different data types curated by WormBase, FlyBase, and MGI, respectively (Additional File 1, Note S1A-B). This method has been incorporated into the curation workflow at WormBase for the past two years. Moreover, we demonstrate a simple automated procedure to combine training papers of similar data types of different databases to train a SVM for the identification of these data types for a single database. This work is potentially very useful as the amount of work necessary for any single database to obtain a sufficient number of training papers for a specific data type, especially those that are found with low occurrence in the literature, may take years.

## Results and Discussion

### Formulation of multi-class problem to categorize multiple curation datatypes

Categorizing curation datatypes is a multi-class problem in which more than two datatypes need to be classified. SVM is a binary classifier; we converted the multi-class problem of the curation datatype to a binary class problem using the one-versus-rest strategy (see Methods).

### Feature selection

We observed that curatable information often resides in a small portion of a document or a few sentences, and rationalized that the frequency of feature usage in a document may not be of significant relevance. This observation was thus taken into consideration for both feature selection in representing the documents and the term weighting scheme for constructing the data vector. We calculated Chi-square scores and mutual information scores as described by Manning *et al. *[24] for all the data types and found that the differences between Chi-square scores of adjacent ranked features were much larger than those of mutual information. We thus think that Chi-square score is a much better criterion for feature selection (data not shown). As shown in Additional File 2, Table S1, many features of the top Chi-square scores for a given data type, for example, RNAi interference, are characteristic of this data type and the same observation was made for other data types as well (data not shown). Along the same line of reasoning, we used a binary scheme to construct a data vector for each of the documents in which a value of 1 is assigned if the feature is present and a value of 0 if not (see Methods).

### Recall and precision measure in biocuration and comprehensive SVM scheme

The performance of an SVM can be evaluated using a testing set containing documents with known class labels. The commonly used evaluation metrics are recall and precision: recall = TP/(TP+FN); precision = TP/(TP+FP); where TP represents true positive, FN represents false negative, and FP represents false positive. A high precision value is normally more readily achievable than high recall in SVM-based text classification [23] and a high precision value has actually been preferred over a high recall in some commonly studied areas such as web page categorization *etc*. In biocuration, however, the goal is to obtain the highest recall possible while keeping the false positive rate reasonably low because, if recall is not high enough, curators would need to examine all published papers for their data type to uncover false negatives. On the other hand, curators would only need to examine a subset of the papers, those identified as positives, to eliminate potential false positives.

To achieve a high recall, we developed a 9-component comprehensive SVM scheme with multiple SVMs using the top 10, 25, 50, 75, 100, 150, 200, 300, and 400 Chi-square score ranked features. We then applied this SVM and calculated the final recall and precision by combining all the papers identified from these SVMs (see Methods). This scheme increased the recall value by as much as ~10% while only causing a tolerable decrease in precision. This comprehensive SVM scheme was also utilized to increase the confidence of the identification (see Methods). Unless indicated otherwise, all the results presented here were analyzed using this comprehensive SVM scheme.

The recall and precision values of each single SVM component as well as the comprehensive SVM analysis were shown in Additional File 3, Table S2. In general, for each component SVM, the recall value is lower than the precision value, and the number of top ranked features required to give the best recall varies in different data types.

The comprehensive SVM analysis generally increased recall and decreased the precision value in comparison to the single component SVMs. The effects are more prominent for some data types than others. For example, in the case of RNAi data, the comprehensive SVM achieved a recall of 0.99, whereas the recall of a single SVM component is 0.91 and the worst recall of single SVM is 0.85. On the other hand, the increase of recall in comprehensive SVM is not so apparent for the antibody data type. The recall of the comprehensive SVM for antibody is 0.94, which is a slight increase from 0.91, the best recall of the single SVM components, and 0.88, the worst recall of the single SVM components.

The decrease in precision in comprehensive SVM also varies with different data types. For example, for the RNAi data type, the precision of comprehensive SVM is 0.78, which is much lower than the best precision of 0.92 of a single component SVM and is also lower than the worst precision of 0.82 of a single component. On the other hand, for the Mutant allele sequence data type, the precision of the comprehensive SVM is 0.98, not much of a decrease in comparison to both the best and the worst precision of a single component SVM, 1 and 0.98, respectively.

It is not clear whether the same single component SVM will give the highest recall in the testing set and different batches of validation set; we do not have sufficient validation sets to do a systematic evaluation. It is thus generally more desirable to do comprehensive SVM analysis to improve recall.

### Automated data type identification for WormBase and FlyBase curation

To test our method, we applied it to ten data types (Additional File 1, Note S1A) of strong interest to WormBase. A sufficient number of papers labeled with these ten data types have accumulated between 1985 - 2009 by curators reading each new *C. elegans *paper and indexing different data types; these labels were used in constructing the training sets. Each paper underwent comprehensive SVM analysis for each of the ten data types (Table 1; Additional File 4, Table S3) and the performance for each data type was evaluated by using a testing set with papers from the same time period as that of the training set, which is from papers curated at WormBase between 1985 and 2009 (see Methods). Six of the data types were also evaluated every one-two weeks using new *C*. e*legans *papers, i.e. the validation sets, over a six-month period (07/2009 - 12/2009) (see Methods). The recall and precision values of these ten data types from the testing set were in the range of 0.85 - 0.99 and 0.70 - 0.98, respectively. The recall and precision values from the validation sets agreed well with those from the testing sets for all the data types except the gene expression and gene regulation data types whose precision values decreased from 0.98 to 0.55 and 0.88 to 0.49, respectively.

**Table 1:** Evaluation results of ten WormBase data types using the ten testing sets

Data types	Recall (testing set)	Precision (testing set)
RNAi	0.99	0.78
Antibody	0.94	0.81
Phenotype	0.86	0.92
Gene regulation	0.88	0.70
Mutant allele sequence	0.93	0.98
Gene expression	0.95	0.88
Gene product interaction*	NA	NA
Overexpression phenotype	0.91	0.81
Gene interaction	0.85	0.79
Gene structure correction	0.90	0.82

The SVM analysis was done using training/testing sets specified in Additional File 4, Table S3 and Methods. *Gene product interaction does not have enough labeled papers and no evaluation was done using the testing set.

The number of papers in each batch varies depending on how many papers on *C. elegans *were published in the relevant time period. For example, for the five batches validated for RNAi data, the number of papers ranged from 19 to 88. The SVM performance for RNAi data type among different batches varied little judging by the standard deviation of recall and precision: recall of these five batches is 0.98 +0.04 and precision is 0.81 ± 0.03. We also examined the precision value of SVM analyses of six batches for gene expression data type. These six batches ranged from 21 to 44 papers, and the average precision value is 0.44 ± 0.08. The performance of a batch was not correlated with its size. For example, the batch with the highest precision (0.59), and the batch with the lowest precision (0.37), have about the same number of papers, 21 and 22, respectively. The precision of the largest batch with 44 papers is 0.45, close to the average.

Several factors may contribute to the decrease in the precision value from the validation set for gene expression and gene regulation data type, in comparison to those from the testing set: Data type definitions may change over time, and different vocabularies may be used to describe data type-specific information as new experimental methods are invented or old experimental methods become obsolete. For example, when looking at gene expression, Northern blotting was commonly used in the past but is now less frequently used, having been replaced by techniques such as reporter gene expression and RT-PCR.

The training papers for gene expression and gene regulation, the data types whose validation set showed much lower precision than the testing set, are obtained from a collection of the past 14 years. We do not have sufficient training papers to make large enough training set for different period of time to examine the time effect; this can be done more effectively at a later time when significant number of newly labeled papers are available for systematic comparison.

The SVM method does not take into account synonym expansion; the change in the vocabulary of the used terms might lead to decreased performance. This type of change may be one of the reasons that the precision of the validation set for gene expression and gene regulation data types are much lower than those from the testing set. This problem can be addressed by utilizing generalized vector space models, or concept vector space models that map terms into concepts, and the document can then be categorized based on concepts which accommodate terms from different times instead of terms that may change over time [34]. It has been shown that the SVM performance in precision was significantly increased especially in those cases with small training sets after incorporating WordNet concepts for mapping the terms [34].

We also applied the comprehensive SVM method to fifteen data types from FlyBase (Additional File 1, Note S1B). Table 2 and Additional File 5, Table S4 show the results of five of these data types with high occurrence. Their performances were similar to those of the WormBase data types with recall in the range of 0.88 - 0.98 and precision in the range of 0.56 - 0.92.

**Table 2:** Evaluation results of Five FlyBase data types with high occurrence using the testing sets

Data type	Recall	Precision
New mutant allele	0.98	0.56
Gene expression in wild-type background	0.96	0.92
Gene expresison in perturbed background	0.95	0.92
New transgenic allele	0.91	0.71
Physical interaction between macro-molecules	0.88	0.84

The SVM analysis was done using training/testing sets specified in Additional File 5, Table S4 and Methods.

### SVM across organism-specific corpora

The same or similar types of data are often curated at different biological databases such as the model organism database, or MODs. For some data types, the training set from one MOD may not be large enough to achieve satisfactory performance. We thus explored the possibility of utilizing training papers from one MOD to help with the SVM analysis of similar data types in another MOD. Both WormBase and FlyBase label papers containing RNA interference (RNAi) data, albeit using different criteria (Additional File 1, Note S1A-B). WormBase has identified > 1400 papers indexed with 'RNAi', while FlyBase has identified only 232 'RNAi'-labeled papers.

One strategy for utilizing the large training set of C. *elegans *papers to identify *D. melanogaster *papers that contain the RNAi data type would be to remove *C. elegans *specific features from the *C. elegans *RNAi feature list. However, while some features such as "Fire", the surname of an author of a highly cited *C. elegans *RNAi reference, seemed to be a likely candidate for removal, others were not so readily apparent. Thus, manually editing an existing features list could be a difficult and time-consuming process.

We categorized the features of a data type to be either organism-independent or organism-dependent. Those organism-independent features found in *C. elegans *RNAi papers could contribute to the SVM analysis of *D. melanogaster *RNAi papers whereas those features only found in *C. elegans *RNAi papers probably would not contribute to the *D. melanogaster *RNAi SVM. We postulated that by pooling the training papers from WormBase and FlyBase and then calculating the Chi-square score for their features, the ranking of organism-independent features would be more favorable than when the Chi-square score was calculated using only WormBase or FlyBase training papers alone. On the other hand, those organism-dependent features would be less favorable than those found using only WormBase or FlyBase training papers alone. As shown in Additional File 2, Table S1, the top-ranked, organism-specific features such as "Fire" and "Timmons," both author names of a highly cited *C. elegans *RNAi reference, disappeared from the top 400 features list of the combined training set, whereas organism-independent features such as RNAi, dsRNA, interference, *etc*. remained as top-ranked features.

As shown in Table 3 and Additional File 6, Table S5, SVM analysis using a training set containing 170 WormBase RNAi and 170 FlyBase RNAi papers effectively increased the recall from 0.81, obtained using the FlyBase training papers alone, to 0.99, while the precision value remained as high as 0.99, indicating that this pooling strategy worked well. A large training set containing 773 WormBase RNAi papers gave a much lower recall of 0.85 but the same precision value of 0.99 for the same FlyBase testing papers.

**Table 3:** Evaluation results of FlyBase RNAi data type using FlyBase or/and WormBase training papers

Training dataset	Recall	Precision
FlyBase RNAi	0.81	1.00
WormBase production RNAi	0.85	0.99
FlyBase+WormBase RNAi	0.99	0.99

The SVM analysis was done using training/testing sets specified in Additional File 6, Table S5 and Methods.

### Performance measure for data type of low occurrence (unbalanced class distribution)

Many data types have low occurrences, i.e., the number of documents containing the specific data type (i.e. positive set) is much smaller than those not containing the specific data type (i.e. negative set) in the document set of interest. This situation is often referred to as an unbalanced class distribution. For these data types the precision measure was inadequate as the precision value could be affected by the size of the negative set [35]. The precision value could be very low while in fact the percentage of false positive identification was not high at all. For example, for a data type with a 1% occurrence, if 2 of 100 papers were classified as positive of which one is true positive and the other is false positive, this would result in a recall of 1 and a very low false positive rate of 1%. Due to the unbalanced class distribution, the precision value would only be 0.5. The number of papers that would need to be examined to uncover the true positives is only two after the SVM analysis, while 100 papers would be needed to uncover the true positives without SVM analysis. Therefore the precision value reflects neither the false positive rate nor the effectiveness of SVM in improving the curation efficiency. If the same recall and false positive rate occurred in a balanced class distribution with 50 as positive and 50 as negative, the precision value would be a much higher value (0.98) which is more in line with the false positive rate and the effectiveness of SVM in increasing curation efficiency. We thus focus on the "filtering term", FT = 100 * (predicted positive papers)/(total papers) = 100*(TP + FP)/(total papers) i.e., FT = (TP+FP)/(TP+FP+TN+FN). For the above example, FT = 100*(1+1)/100 = 2%, a better indicator of the improvement in the curation efficiency by filtering out negative papers. The lower the FT value, the lower the fraction of papers that need to be examined after filtering by the SVM analysis and thus the higher the improvement in curation efficiency.

### SVM results of data types of low occurrence

Table 4 and Additional File 7, Table S6 show the SVM results of nine data types from FlyBase. Table 5 and Additional File 8, Table S7 show the SVM results of three data types used for the text classification task at the Genomic Track of the Text Retrieval Conference 2005 (GT TREC 2005), which were originally curated by Mouse Genomics Informatics (MGI) [36]. These data types have unbalanced class distributions whose percentage in the total document set were in the range of ~1-10%. It has been reported that a large negative training set can have adverse effects on performance [21,37-39], and several approaches, including modifying either the data distribution or the classifier, or a combination of both, have been applied to deal with this problem [21,37-39] (http://research.microsoft.com/pubs/70007/tr-2003-34.pdf). We found that a large negative training set could have both positive and negative consequences: on the one hand, it could increase precision while on the other hand, it could decrease recall (data not shown). An optimum ratio of positive to negative training sets (PN ratio) could be found for each data type to give the highest recall possible while keeping the false positive rate reasonably low, i.e., a reasonably low filter term (FT) value. As shown in Tables 4 and 5, the recall values for these data types were in the range of 0.86 ± 0.06 to 0.98 ± 0.01 and the filter term (FT) values were between 3.4 ± 1.6% to 22.5 ± 2.3%. The use of the optimum PN ratio effectively increased recall values of these data types from a range of 0.32 - 0.7 to a range of 0.87 - 0.97 while FT values were kept under ~20%.

**Table 4:** Evaluation results of nine FlyBase data types With low occurrence using the testing sets

Data type	Recall	Filter term (%)
Initial characterization of a gene	0.97 ± 0.05	18.0 ± 1.3
Use of expression marker	0.95 ± 0.06	22.5 ± 2.3
Transfection of DNA/RNA	0.94 ± 0.04	7.6 ± 1.6
New phenotype (characterization)	0.93 ± 0.05	19.9 ± 2.1
Renaming of a gene	0.91 ± 0.10	10.9 ± 2.6
New cis-regulatory elements	0.88 ± 0.05	8.1 ± 2.2
Gene model modification	0.88 ± 0.08	17.1 ± 3.5
Genome feature sequence mapping	0.87 ± 0.09	10.9 ± 2.6
Merge of gene reports	0.86 ± 0.06	13.7 ± 5.3

The SVM analysis was done using training/testing sets specified in Additional File 7, Table S6 and Methods.

**Table 5:** Evaluation results of three data types with low occurrence from MGI using the testing sets

Data type	Recall	Filter term (%)
Mutant Phenotype allele	0.98 ± 0.01	12.6 ± 1.2
Embryologic expression	0.94 ± 0.04	11.4 ± 1.7
Tumor biology	0.90 ± 0.08	3.4 ± 1.6

The SVM analysis was done using training/testing sets specified in Additional File 8, Table S7 and Methods.

TF-IDF (Term of Frequency Inverse Document Frequency) is one of the most commonly used term weighting schemes in information retrieval and text mining. We compared SVM analyses using the following three different feature selection methods and term weighting schemes: TF-IDF weighting on all features, TF-IDF weighting on Chi-square score ranked features, Boolean weighting on Chi-square score ranked features using the RNAi data type. The results were evaluated using two testing sets and two validation sets, respectively. The two testing sets differ in the ratio of the negative set of the positive set, one with a 1:1 and the other with a 2:1 ratio, as do the two validation sets. Because the TF-IDF weighting scheme without feature selection is CPU-intensive with large datasets, these comparisons were done using small training and testing sets (Additional File 9, Table S8, Additional File 10, Table S9; Additional File 11, Table S10; and Additional File 12, Table S11), which were constructed by randomly selecting papers from the positive and negative labeled pools. All the different schemes used the same training, testing and validation sets.

The reason we used different ratios to evaluate the results is that we are interested to know how different ratios might affect the evaluation of results. This issue arises because in the curation process, we need to do text categorization of newly published papers on a frequent basis. The ratio of the positive papers over the negative papers in such short period of time could vary batch by batch for any data type and it could differ from the ratio of the training set.

As shown in Additional File 9, Table S8, Additional File 10, Table S9, Additional File 11, Table S10, and Additional File 12, Table S11, Boolean and TF-IDF weighting schemes that combine Chi-Square score ranked feature selection have similar recall, ≥ 0.9. By contrast, TF-IDF weighting schemes using all features (without the feature selection step) have very poor recall, between 0.08 - 0.61. As shown in Additional File 9, Table S8, the TF-IDF weighting scheme that combines Chi-Square feature selection has similar precision as that of the Boolean one when using the testing set with the ratio of negative over positive set of 1:1. In the testing set with a 2:1 ratio of negatives to positives and both the validation sets, a TF-IDF weighting scheme that combines Chi-Squared score ranked feature selection has much lower precision than the Boolean weighting scheme that combines a Chi-Squared score ranked feature selection. As shown in Additional File 10, Table S9, in the validation set with a 1:1 ratio of negatives to positives, the precision of the TF-IDF one is 0.61 whereas the Boolean one is 0.72. As shown in Additional File 11, Table S10, in the testing set with a 2:1 ratio of negatives to positives, the precision of the TF-IDF one is 0.54 whereas the Boolean one is 0.64. As shown in Additional File 12, Table S11, in the validation set with a 2:1 ratio of negatives to positives, the precision of the TF-IDF one is 0.45, whereas the Boolean one is 0.59. The TF-IDF weighting scheme that combines all features gives similar precision values as those of the Boolean weighting scheme that combines Chi-Square score ranked feature selection in all four evaluation sets.

The precision values of the SVM analysis using the TF-IDF weighting scheme are 0.10-0.15 lower than that using the Boolean weighting scheme in three out of four cases reported here. This difference may be due to the fact that the ratio of negative over positive papers in a small pool of new papers can deviate from that of the training set. The TF-IDF may also cause inappropriate scaling for some features; consequently some features with strong predicting power may be given less favourable score than those with weak predicting power, thereby undermining the performance [40]. The ratio of negative papers over the positive papers in each batch of new papers varies and is difficult to predict ahead of time. We think that the Boolean weighting scheme that combines Chi-Square score ranked feature selection maybe a more suitable method than the TF-IDF weighting scheme that combines Chi-Square score ranked feature selection for the categorization of experimental datatypes in a curation process where a small pool of new papers usually need to be analyzed in a timely manner.

Numerous machine-learning methods have been used by various groups that participated in the text categorization task in the GT TREC 2005 challenge [8]. The methods included regularized linear classifier [41], logistic regression [42], pattern-based learning [43], naÏve Bayes learning [44], theme detection [45], K-nearest neighbor [43-45], Rocchio-based classifier [45], SVM [42,44-47], as well as others. Several groups have used SVM in their studies on these data types and have reported different performances. The differences in performance might arise from the use of different feature selection strategies and other procedures in their SVM analysis [36]. One of the SVM method submitted to TREC 2005 has an overall high performance in a comparison with all the other methods submitted [48]. We did a side-by-side comparison of our method and those methods submitted to the GT TREC 2005 for the categorization of the Mutant Phenotype Alleles, Embryologic Expression and Tumor Biology data types [8,48] originally curated by MGI. As shown in Additional File 10, Table S9, our method showed equivalent or better results for all the three data types than both the best performance among various methods and a SVM method submitted to the GT TREC 2005. In comparison to the best performance among various methods submitted to GT TREC 2005 [48], our method achieved similar recall for all three data types and a 1.3- and 2.4-fold increase in precision for the Mutant phenotype allele and the Tumor biology data type, respectively. In comparison to the SVM method submitted to the GT TREC 2005 [48], our method gave a higher recall value, 0.94 ± 0.04, compared to 0.82, and a similar precision value for the Embryologic expression data type. For the other two data types, our method gave similar recall but more than 2-fold increase in precision. Furthermore, our method is relatively simple when compared to most of the methods submitted to GT TREC 2005, which involved multiple steps or required expert domain knowledge in feature selection or document preprocessing etc. Our method does not require any data type specific manual input or sophisticated manipulation at any step, is completely automated, and can be readily applied to different data types.

We showed that our method can be applied to the three data types of MGI giving high recall (Additional File 13, Table S12), and thus might save curation time (measured by the FT term). However, a direct comparison of our method and those methods in TREC 2005 is difficult because we used a different set and number of papers for training and testing (Additional File 10, Table S9) than those used by TREC 2005 participants. As indicated earlier, the PN ratio affects precision value. In the TREC 2005 systems, the number of negative training papers is much larger than that of the positive papers: this disparity may adversely affect precision. We think that this factor may need to be taken into consideration when evaluation schemes are designed.

Previously we developed a combinatorial Boolean keyword search using Textpresso [44] to identify papers that contained the RNAi data type (G. Schindelman, J. Chan, and P. Sternberg, unpublished results) with a recall of 0.96 and precision of 0.61. This was obtained after eight iterations of refining keywords in the search query and subsequent manual examination of false negative and false positive articles. This process requires expert domain knowledge for a specific data type and time consuming manual effort, unlike the SVM method which is completely automatic with a given training set and can be readily used for different data types. Furthermore, for those data types without a sufficient set of specific keywords, this approach may not be applicable.

Once documents have been classified for data type identification, a subsequent task in biocuration is extraction of the information of interest. While attempts to automate fact extraction can be undermined by high false positive rates, we have observed that the false positive rate in text extraction of Gene Ontology Cellular Component data by a category-based semi-automatic text extraction approach using Textpresso [14] is significantly decreased when extraction is performed on only those papers identified as containing gene expression data by SVM (K. Van Auken, R. Fang, J. Chan, H.-M. Müller, and P. Sternberg, unpublished results). We expect that a filtering step provided by SVM analysis will have the same effect on other text extraction methods, as well.

## Conclusions

Although the SVM algorithm has been successfully applied to text classification for nearly 20 years, its use in categorizing bioscience literature has been limited to specific cases [49]. We present here a methodology for its successful application for a broad range of data types as specified by the following three main points. First, Chi-square scores appear to be a suitable criterion for feature selection in the classification of diverse data types in biocuration. Second, training papers of similar data type from different databases (such as different MODs) can be pooled to train SVM for successful identification of a similar data type for different databases. This is especially useful for those data types of low occurrence as it could take a long period of time for each individual database to collect sufficient training papers. Third, for data types with unbalanced class distribution, desirable performance can be achieved by using a suitable PN ratio that could be readily implemented for different data types. Most studies concerning data with unbalanced class distribution have concentrated on those cases with extremely unbalanced class distribution, and there has not been much systematic study of how different levels of unbalance in the class distribution may affect SVM performance in different application fields. We have observed that PN ratio affected performance even with some data types of relatively high occurrence and that the composition of the negative training set also has effects on the performance (data not shown). A systematic and thorough examination in the future may provide more insight for better utilization of SVM algorithms for text classification. The method presented here can be readily adopted by different biological databases for automatic identification of papers of diverse data types, thereby greatly reducing time spent on an otherwise laborious and demanding task [49,50]. We anticipate that the work and observations described herein will help not only biological databases with their curation, but also text mining researchers to improve existing, or develop better, text classification algorithms.

## Methods

### Document pre-processing

For those data types from WormBase, WormBase IDs and PMIDs of papers for use in training/test sets or new incoming papers were obtained from WormBase or an in-house curation status tracking database (J. Chan and P. Sternberg, unpublished data). For those data types from FlyBase, FlyBase IDs and PMIDs of labeled papers were provided by FlyBase. The negative examples for both WormBase and FlyBase were a collection of papers labeled as not containing any of the curatable data types. The negative set for a particular data type was then constructed by combining this negative set of papers and papers that are positive for all other data types. This negative set is not ideal as the true negative set should be all the *C. elegans *papers minus the papers of the particular data type under consideration. For data types from the GT TREC 2005, PMIDs of positive and negative sets were obtained as indicated at the website (http://ir.ohsu.edu/genomics). Papers were downloaded, and converted to full text versions, including references, using a wrapper Perl script of the pdf to text conversion library pdftotext [51,52].

### An SVM classification scheme for multi-class curation datatypes

The categorization of curation data types is a multi-class problem in which more than two data types need to be classified. A paper is labeled as containing a data type if it contains only the data of the given data type or if it contains the data of the given data type and any other data types. SVM is a binary classifier, and to use this efficient method, we converted the multi-class problem of the curation data type to a binary class problem using the one-versus-rest strategy. For example, to categorize RNAi data type, we run SVM analysis to classify RNAi and non-RNAi papers. For the gene expression data type, we run SVM analysis to classify gene regulation and non-gene regulation papers and so forth. Every paper is analyzed for every data type. A paper can have a single label if it only contains one data type of curation interest, or it can have multiple labels if it contains more than one datatypes of interest.

### Construction of training and testing set

For the ten data types from WormBase, training and testing sets were constructed according to the numbers listed in Additional File 4, Table S3 using a labeled paper collection in the period of 1985 - 2009 at WormBase. Briefly, for those data types with a sufficient number of labeled positive papers, the datasets were split into training and testing sets by the following procedure: the positive and negative labeled papers were sorted accorded according to their WormBase PaperID, which was assigned on a chronological order. Those with odd order number are assigned to the training pool and those with even order number were assigned to the testing pool. In this well controlled experiment, where the testing set is very similar to the training set, we could quickly evaluate whether the training set is large enough to achieve good performance and whether SVM works for a particular data type at all. Once this was established, we could evaluate the results using real-life examples, which are the current papers to see whether this method is applicable to our curation process. For those data types with a limited number of labeled positive papers, a small fraction of papers were randomly selected for testing and the remaining were used for training.

For the five data types with high occurrences from FlyBase, training and testing sets were constructed according to the numbers listed in Additional File 5, Table S4. Both positive and negative training and testing sets were randomly selected from their respective labeled pools. For the nine data types with low occurrences from FlyBase and three data types with low occurrence from MGI, due to the limited number of positive labeled papers, a small portion (10-45 papers) was randomly selected to make up the positive testing set and the remaining larger portion was kept to make up the positive training set. To avoid possible bias caused by the small testing set, ten different positive training testing sets were constructed by such random selection process. The negative training and testing sets were constructed in similar fashion. The results shown are the average of these ten data sets.

To construct the training and testing set with different positive:negative (PN) ratio of positive training set over negative training sets for the three MGI data types, the one positive training set was constructed by randomly select positive papers from the positively labeled papers. For the same positive training set, different number of negative papers were randomly selected so that the ratio between the positive and the negative training set are 1:1, 1:1.5, 1:2, 1:3, 1:6. Comprehensive SVM analysis was then conducted on each of the training/testing pair and only the best performing are reported in Table 5 and Additional File 8, Table S7.

### Construction of validation set for WormBase data types

Validation data set for WormBase SVM analysis were *C*. e*legans *papers published over a six months period (07/2009 - 12/2009). To identify whether a paper is *C*. e*legans *paper, a key word containing elegans were used to search all the abstracts on PubMed and those abstract returned were then manually examined to determine whether the publication is new research on *C. elegans*. For those publications with new research work on *C. elegans*, their full pdf files were then manually downloaded. The SVM analysis on our curation production line where the validation sets were taken from was normally done on ~ bi-weekly and sometimes monthly basis. The number of papers in each batch varies depending how many *C. elegans *papers were published in that time period and it could range from ~20 - ~100. Supplementary material was also analyzed since experimental information is sometimes mentioned in the supplementary material but not in the full text.

### Feature selection and the construction of data vectors

For each pair of the positive and negative training sets for each data type, their features were extracted and the corresponding Chi-square scores were calculated as described by Manning *et al *[24]. Nine feature lists consisting of the top 10, 25, 50, 75, 100, 150, 200, 300, 400 features respectively were constructed for each data type. A data vector for each document with each feature list was constructed using a binary scheme where 1 was assigned if the feature from the feature list was present in the document and 0 if not.

### Filtering Term (FT)

For data types with low occurrence, i.e., the number of documents containing the specific data type (i.e. positive set) is much smaller than those documents not containing the specific data type (i.e. negative set) in the document set of interest, the precision measure was inadequate as it can neither reflects the false positive rate nor the effectiveness of SVM in improving the curation efficiency. We thus define a filtering term, FT = 100 * (positive papers)/(total papers) = 100*(TP + FP)/(total papers) = (TP+FP)/(TP+FP+TN+FN). This filtering term reflects the improvement of curation efficiency of SVM analysis by filtering out the negatives identified by SVM. The lower the FT term, the fewer papers are in the positive pool and fewer false positive papers need to be examined.

### SVM library

We chose LIBSVM [33] as it includes a utility for data set scaling, 5-fold cross-validation, and the optimization of SVM parameters (http://www.csie/ntu.edu.tw/~clin/libsvm). The Radial Basis Function (RBF) kernel was used as recommended by the LIBSVM user guide (http://www.csie.ntu.edu.tw/~cjlin/papers/guide/guide.pdf). Some users have noted that LIBSVM is very slow with large datasets, whereas SVM-Light has performed well with very large datasets [32] (http://svmlight.joachims.org). For our work, all datasets were in the small-to-medium range.

### Confidence of comprehensive SVM

We assigned an empirical confidence scheme where a confidence level of low, medium, and high was given if a paper was found to be positive in 1-3, 4-6, or 7-9 SVMs respectively. A cutoff at high, medium, or low level can be applied to obtain the most desirable combination of recall and precision value for the users. The higher the cut-off is, the higher the precision and the lower the recall is respectively.

### Computer programs

All programs were written in Perl and Python and are available for download (Additional File 14, easySVM.tar.gz).

## Authors' contributions

RF developed the algorithm, wrote the program and analyzed all the datasets. GS contributed to the comprehensive SVM scheme and validated RNAi results. KVA validated gene product (GO) and mutant allele sequence results. JF validated phenotype analysis results. WC validated gene expression and antibody results. XW validated gene regulation results. PD validated the training set used for the gene structure correction data type. MAT validated mutant allele sequence results. SM and GM provided the FlyBase Cambridge datasets. BM and HZ provided the FlyBase Harvard datasets. RF wrote the paper with valuable discussion and critical contributions at all stages of the project from PWS. PWS, GS, KVA, GM, BM, and SM edited the manuscript. All authors read and approved the final manuscript.

## Supplementary Material

Additional file 1 **Figure S1. Diagram of a linear Support Vector Machine; Note S1. Definition of Data Types**. Click here for file

Additional file 2 **Table S1. Top 400 Chi-square score ranked features for the RNAi data type**. Top 400 Chi-square score ranked features and their corresponding Chi-square scores for the RNAi data type determined using an RNAi training set of 773 positive and 2543 negative WormBase papers, an RNAi training set of 170 positive and 1100 negative papers, an RNAi training set of 170 positive and 1044 negative FlyBase papers, and an RNAi training set of 170 positive FlyBase + 170 positive WormBase papers and 1044 negative FlyBase + 1100 negative WormBase papers are listed under the following columns respectively: Feature_773WB (Features using 773 WormBase RNAi training papers); χ^2^_773WB (Chi-square scores using 773 WormBase RNAi training papers); Feature_170WB (Features using 170 WormBase RNAi training papers); χ^2^_170WB (Chi-square scores using 170 WormBase RNAi training papers); Feature_170FB (Features using 170 FlyBase RNAi training papers); χ^2^_170FB (Chi-square scores using 170 FlyBase RNAi training papers); Feature_170WB+170FB (Features using 170 WormBase + 170 FlyBase RNAi training papers); χ^2^_170WB+170FB (Chi-square scores using 170 WormBase + 170 FlyBase RNAi training papers). Those Features in bold red are examples of organism-dependent features and those features in bold blue are examples of organism-independent features. Chi-square scores were calculated as described by Manning *et al. *[24] Click here for file

Additional file 3 **Table S2. Comparison of recall and precision of single SVM component and comprehensive SVM**. This table shows the results of SVM analyses using top 10, 25, 50, 75, 100, 150, 200, 300, 400 Chi-Square score ranked features respectively and the comprehensive results of these SVM analyses. The runs with the best and worst recalls were highlighted in blue, bold and green, bold, respectively, and the results of the comprehensive SVM were highlighted in red, bold. Click here for file

Additional file 4 **Table S3. Evaluation results of ten WormBase data types using the validation sets**. The SVM was done in the same way as that in Table 2. Those recall values labeled by * were estimated by examining the false negatives in twenty randomly selected negatives after SVM analysis. Recall and precision values from the validation sets were the average of the results from the validation batches listed which were conducted on a weekly to bi-weekly basis on the new incoming papers over a six month period (07/2009 - 12/2009). Click here for file

Additional file 5 **Table S4. Evaluation results of five FlyBase data types with high occurrence using the testing sets**. This table extends Table 2 with several additional columns, showing further information of the data set used in the SVM analysis. Click here for file

Additional file 6 **Table S5. Evaluation results of FlyBase RNAi data type using FlyBase and/or WormBase training papers**. This table extends Table 3 with several additional columns, showing further information of the data set used in the SVM analysis. Click here for file

Additional file 7 **Table S6. Evaluation results of nine FlyBase data types with low occurrence using the testing sets**. This table extends Table 4 with additional columns, showing further information of the datasets used in the SVM analysis. Click here for file

Additional file 8 **Table S7. Evaluation results of three data types with low occurrence from MGI using testing set**. This table extends Table 5 with several additional columns, showing further information of the datasets used in the SVM analysis. Click here for file

Additional file 9 **Table S8. Comparison of comprehensive SVM analysis using TF-IDF weighting scheme with all features (without feature selection), TF-IDF weighting scheme that combines Chi-Square score ranked feature selection and Boolean weighting scheme that combines Chi-Square score ranked feature selection: first testing set**. The same training and testing sets were used for all three analysis schemes. The training set consists of 289 positive and 289 negative papers that were randomly selected from the positive and negative labeled pools, respectively. The results were evaluated using two testing sets and two validation sets (Tables S9-S11). This Table shows the results of the first testing set with 124 positive and 124 negative papers. All the positive and negative testing and validation sets were randomly selected from their respectively labeled pools as described in Methods. Click here for file

Additional file 10 **Table S9. Comparison of comprehensive SVM analysis using TF-IDF weighting scheme with all features (without feature selection), TF-IDF weighting scheme that combines Chi-Square score ranked feature selection and Boolean weighting scheme that combines Chi-Square score ranked feature selection: first validation set**. The same training and testing sets were used for all three analysis schemes. The training set consists of 289 positive and 289 negative papers that were randomly selected from the positive and negative labeled pools, respectively. The results were evaluated using two testing sets and two validation sets (Tables S9-S11). This Table shows the results of the first validation set with 49 positive and 49 negative papers. All the positive and negative testing and validation sets were randomly selected from their respectively labeled pools as described in Methods. Click here for file

Additional file 11 **Table S10. Comparison of comprehensive SVM analysis using TF-IDF weighting scheme with all features (without feature selection), TF-IDF weighting scheme that combines Chi-Square score ranked feature selection and Boolean weighting scheme that combines Chi-Square score ranked feature selection: second testing set**. The same training and testing sets were used for all three analysis schemes. The training set consists of 289 positive and 289 negative papers that were randomly selected from the positive and negative labeled pools, respectively. The results were evaluated using two testing sets and two validation sets (Tables S9-S11). This Table shows the results of the second testing set with 62 positive and 124 negative testing papers. All the positive and negative testing and validation sets were randomly selected from their respectively labeled pools as described in Methods. Click here for file

Additional file 12 **Table S11. Comparison of comprehensive SVM analysis using TF-IDF weighting scheme with all features (without feature selection), TF-IDF weighting scheme that combines Chi-Square score ranked feature selection and Boolean weighting scheme that combines Chi-Square score ranked feature selection: second validation set**. The same training and testing sets were used for all three analysis schemes. The training set consists of 289 positive and 289 negative papers that were randomly selected from the positive and negative labeled pools, respectively. The results were evaluated using two testing sets and two validation sets (Tables S9-S11). This Table shows the results of the validation set with 49 positive and 98 negative validation papers, respectively. All the positive and negative testing and validation sets were randomly selected from their respectively labeled pools as described in Methods. Click here for file

Additional file 13 **Table S12. Comparison of our SVM classification results with those from TREC 2005**. This table compared the performance using our Boolean weighting scheme that combines the Chi-Square ranked feature selection SVM method with that of the best performed machine learning method and a SVM method from TREC 2005 for Mutant phenotype allele, Embryologic expression and Tumor biology data types from MGI. Click here for file

Additional file 14 **easySVM.tar.gz**. This file contains instructions, code and samples set for text categorization described here. Please read the file named INSTRUCTION under the directory easySVM after decompressing the tar ball before running the analyses. Click here for file
