# Supplementary material for: Automatic categorization of diverse experimental information in the bioscience literature
Source: BMC Bioinformatics. 2012 Jan 26;13:16. doi: 10.1186/1471-2105-13-16 (PMC3305665; doi:10.1186/1471-2105-13-16)
Supplement: Additional file 1 — Figure S1. Diagram of a linear Support Vector Machine; Note S1. Definition of Data Types. [file 1471-2105-13-16-S1.PDF]

Supplementary Figure 1. Diagram of a Linear Support Vector Machine.

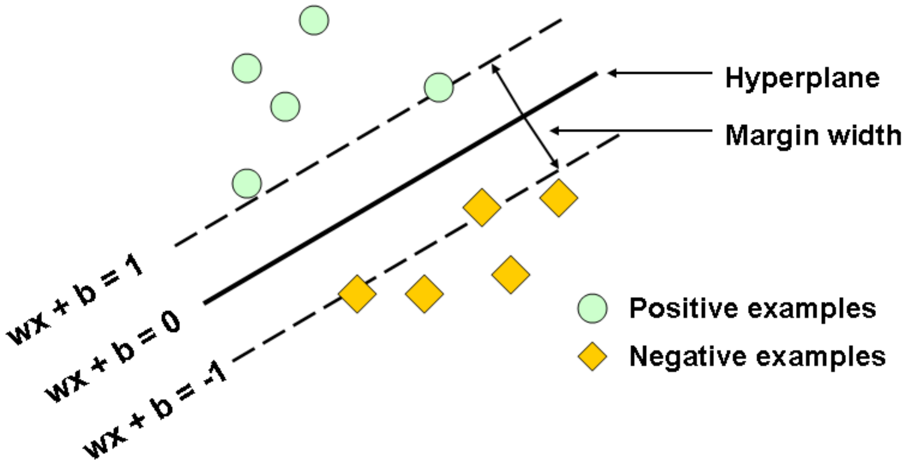

Supplementary Note 1

A. Definition of data types from WormBase

a. **RNAi**: Gene function assayed by RNA interference.

b. **Antibody**: Antibody generated in a noncommercial laboratory, against a *C. elegans* gene product.

c. **Phenotype**: Phenotypes of mutants or phenotypic analysis of strains.

d. **Gene regulation**: Changes or a lack of changes in gene expression levels or patterns in response to genetic, chemical, temperature, or an other experimental treatment.

e. **Mutant allele sequence**: Sequence data for any mutation.

f. **Gene expression**: New temporal or spatial (*e.g*., tissue, subcellular, *etc*.) data on the pattern of expression of any gene in a wild-type background, this data type includes reporter gene analysis, antibody staining, *in situ* hybridization, RT-PCR, Western or Northern blot data.

g. **Gene product interaction**: A subset of gene product interactions that contains only those papers that describe macromolecular interactions.

h. **Overexpression phenotype**: Phenotypes due to the overexpression of transgenes.

i. **Gene interaction**: Genetic interactions: genes assayed for effect on the function of another gene. Often this is made apparent by the

analysis of double, triple, *etc*. mutants, or with the use of experiments where RNAi has been used concurrently with other RNAi-treatment(s) or mutations.

j. **Gene structure correction**: The paper reports a gene structure that is different from the one in WormBase, *e.g.*, different splice-site, SL1 instead of SL2, *etc*.

B. Definition of data types from FlyBase

a. **Initial characterization of a gene**: The authors indicate that their paper represents the first significant characterization of a Drosophilid gene or natural transposable element.

b. **FlyBase RNAi**: The paper includes experiment(s) using RNAi against a *D. melanogaster* gene, conducted either in cell culture or transiently in the whole organism.

c. **Gene expression in perturbed background**: The paper presents *D. melanogaster* gene expression data assayed in a genetically/chemically/environmentally perturbed background.

d. **Gene expression in wild-type background**: Either (i) the authors indicate that they are reporting a "novel" expression pattern for a *D. melanogaster* gene assayed in a wild-type background; and/or (ii) the paper includes a "comprehensive" description of the expression pattern for a *D. melanogaster* gene assayed in a wild-type

background. Expression may be assayed by transcript or protein, and be *in situ* or *in vitro*.

e. **Genome feature sequence mapping**: The paper maps alleles, insertions, rescue fragments, or aberration breakpoints to the *D. melanogaster* genome at the molecular level (sequence-based or restriction fragment-based), such that this information can be layered onto the *D. melanogaster* genome annotation.

f. **Merge of gene reports**: The paper indicates that two or more Genes or Natural Transposable Element Reports need to be merged.

g. **New cis-regulatory elements**: The paper includes new experimental data defining cis-regulatory elements of *D. melanogaster* genes, such as enhancers or boundary elements..

h. **Gene model modification**: The paper includes new experimental data relevant to *D. melanogaster* gene model structure, such as the correction of an existing gene model or the discovery of new splice variants.

i. **New mutant allele**: The authors state that they have generated a new classical (as opposed to transgenic) mutant allele of a Drosophilid gene, or that they have generated a new aberration within a Drosophilid genome.

j. **New phenotype (characterization)**: Either (i) the authors indicate that they are reporting “novel” phenotypes associated with a

Drosophilid gene; and/or (ii) the paper includes a “comprehensive” phenotypic characterization of one or more Drosophilid genes.

k. **New transgenic allele**: The authors state that they have generated a new transgenic (as opposed to classical) allele of a Drosophilid gene. For example: UAS transgenes, genomic rescue transgenes, promoter fusion transgenes.

l. **Physical interaction between macro-molecules**: The paper includes experiments investigating physical interactions involving *D. melanogaster* proteins and/or nucleic acids, such as:
    i. Protein-protein interactions *e.g*. yeast hybrid, co-IP.
    ii. Protein-nucleic acid interactions *e.g*. footprinting, DNA/RNA binding.
    iii. Interactions between complexes and other things *e.g*. microtubule binding.

m. **Renaming of a gene**: The authors suggest a renaming of a gene or natural transposable element.

n. **Transfection of DNA/RNA**: The paper includes experiment(s) in which *D. melanogaster* DNA/RNA is transfected (stably or transiently) into cultured cells of Drosophila origin.

o. **Use of expression marker**: The paper includes use of an expression marker (assayed by any method: RNA/protein levels, reporter transgene *etc*.) for a particular *D. melanogaster* cell type/tissue/structure.

C. Definition of data types from Mouse Genomics Informatics (MGI)

a. **Alleles of mutant phenotypes**

b. **Embryologic gene expression**

c. **Tumor biology**
