# Supplementary material for: Automatic categorization of diverse experimental information in the bioscience literature
Source: BMC Bioinformatics. 2012 Jan 26;13:16. doi: 10.1186/1471-2105-13-16 (PMC3305665; doi:10.1186/1471-2105-13-16)
Supplement: Additional file 2 — Table S1. Top 400 Chi-square score ranked features for the RNAi data type. Top 400 Chi-square score ranked features and their corresponding Chi-square scores for the RNAi data type determined using an RNAi training set of 773 positive and 2543 negative WormBase papers, an RNAi training set of 170 positive and 1100 negative papers, an RNAi training set of 170 positive and 1044 negative FlyBase papers, and an RNAi training set of 170 positive FlyBase + 170 positive WormBase papers and 1044 negative FlyBase + 1100 negative WormBase papers are listed under the following columns respectively: Feature_773WB (Features using 773 WormBase RNAi training papers); χ2_773WB (Chi-square scores using 773 WormBase RNAi training papers); Feature_170WB (Features using 170 WormBase RNAi training papers); χ2_170WB (Chi-square scores using 170 WormBase RNAi training papers); Feature_170FB (Features using 170 FlyBase RNAi training papers); χ2_170FB (Chi-square scores using 170 FlyBase RNAi training papers); Feature_170WB+170FB (Features using 170 WormBase + 170 FlyBase RNAi training papers); χ2_170WB+170FB (Chi-square scores using 170 WormBase + 170 FlyBase RNAi training papers). Those Features in bold red are examples of organism-dependent features and those features in bold blue are examples of organism-independent features. Chi-square scores were calculated as described by Manning et al. [24] [file 1471-2105-13-16-S2.PDF]

Feature_773WB: Features using 773 WormBase RNAi training papers.
χ2_773WB: Chi-square scores using 773 WormBase RNAi training papers.
Feature_170WB: using 170 WormBase RNAi training papers.
χ2_170WB: Chi-square scores using 170 WormBase RNAi training papers.
Feature_170FB: Features using 170 FlyBase RNAi training papers.
χ^2_170FB: Chi-square scores using 170 FlyBase RNAi training papers.
Feature_170WB+170FB: Features using 170 WormBase + 170 FlyBase RNAi training papers.
χ2_170WB+170FB: Chi-square scores using 170 WormBase + 170 FlyBase RNAi training papers.

Feature_773WB	χ2_773WB	Feature_170WB	χ2_170WB	Feature_170FB	χ^2_170FB	Feature_170WB+170FB	χ2_170WB+170FB
dsRNA	1093.9	**dsRNA**	428.5	**dsRNA**	394.6	**RNAi**	1110.1
RNAi	1082.1	**RNAi**	416.0	**RNAi**	368.8	**dsRNA**	1030.1
interference	828.8	**Timmons**	311.1	knockdown	215.2	**interference**	754.0
stranded	669.9	**interference**	296.4	dsRNAs	210.9	stranded	693.7
HT115	616.0	HT115	277.7	stranded	186.0	RNA	609.7
Fire	592.2	dsRNAs	269.0	**interference**	171.5	dsRNAs	564.0
Timmons	554.4	L4440	221.0	Depletion	164.1	knockdown	480.5
L4440	515.5	stranded	205.5	Ambion	126.6	double	475.2
Ahringer	451.2	Ingestion	164.0	transfected	124.3	antibodies	442.4
Montgomery	431.3	Kostas	162.3	S2	123.9	depletion	425.7
N2	428.9	Court	157.6	depleted	116.9	treated	423.9
Driver	428.9	Driver	157.1	knocked	108.8	S2	418.5
RNA	426.8	Kamath	154.8	yes	108.0	antibody	418.0
dsRNAs	423.3	ingested	153.0	depletion	107.7	Drosophila	417.1
Kostas	415.7	Zipperlen	152.0	plated	105.7	embryos	410.4
injected	395.5	**Fire**	148.9	treated	100.9	anti	409.5
Kamath	385.3	Potent	144.5	transfection	91.4	depleted	403.6
L4	384.7	lethality	143.7	6499	89.0	cells	399.7
images	384.4	RNA	141.4	6503	89.0	control	390.1
T7	373.2	Ahringer	140.2	culture	88.6	proteins	389.2
GFP	372.5	Embryos	133.5	FBS	87.9	stained	385.2
double	370.7	Montgomery	130.4	streptomycin	83.1	Depletion	385.2
Embryos	364.0	feeding	128.7	coverslips	82.5	mediated	383.6
progeny	359.5	Campos	123.9	T7	82.5	required	380.3
lethality	356.4	Interference	120.9	treatment	82.0	experiments	379.7
Mello	356.2	103112	115.7	Invitrogen	81.5	conserved	376.3
Zipperlen	354.2	embryo	112.4	penicillin	78.7	staining	374.7

Potent	350.0	854	111.2	MEGAscript	73.5	role	374.5
feeding	341.0	double	108.0	Maehama	73.5	pathway	373.6
pPD129	317.1	division	105.6	2007	72.5	expressing	368.8
scored	317.1	embryonic	103.2	knockdowns	71.7	regulate	368.8
nuclei	312.3	mitotic	102.9	Cells	71.6	functions	367.3
hermaphrodites	312.2	embryos	100.5	supplemented	70.0	dependent	367.1
strains	310.7	sterility	97.9	lysed	66.1	function	366.8
embryos	306.3	IPTG	96.4	untreated	65.1	defects	366.7
embryonic	302.7	cytokinesis	96.4	RNA	63.9	cell	366.6
gonad	298.5	Fraser	95.9	Clemens	63.4	protein	365.9
ingested	293.7	391	93.8	Vale	63.2	during	365.2
DIC	293.6	spindle	93.1	Hemmings	62.8	previously	364.6
stained	290.1	bacterially	93.0	kinetochores	62.8	GFP	364.0
Fraser	287.8	L4	92.9	resuspended	61.6	mammalian	363.8
Campos	286.8	phenotype	92.6	pMT	60.4	did	361.3
Court	283.3	phenotypes	92.3	identifi	60.1	roles	361.2
Kohara	282.7	embryogenesis	91.9	Megascript	60.1	binding	360.0
IPTG	279.3	Poulin	90.5	Maiato	60.1	after	358.0
staining	275.5	gonad	89.3	Schneider	58.8	reduced	355.2
embryogenesis	270.7	defects	87.4	western	57.4	we	354.4
embryo	266.7	DAPI	87.4	cultured	56.7	treatment	353.0
phenotypes	266.1	lethal	86.1	Schneiders	55.5	regulation	352.6
gonads	265.9	hermaphrodites	85.8	targeting	55.3	We	351.9
arrowheads	260.7	Mello	85.4	Muda	54.0	performed	351.5
391	260.7	Martinez	85.2	Leff	54.0	0	351.3
strain	260.5	pie	85.0	Wollman	53.2	described	350.9
Effectiveness	259.1	Bowerman	84.4	rupress	52.5	nuclear	349.3
PCR	257.6	Severson	84.0	nal	51.8	gene	349.2
Strains	254.8	Gotta	83.5	Cellfectin	51.5	phenotypes	348.4
injection	254.6	anaphase	83.0	kinetochore	51.2	activity	346.5
phenotype	252.9	divisions	82.8	dsRNAi	50.1	used	345.3
Ingestion	251.4	Effectiveness	82.7	Identifi	50.1	complex	345.2
Xu	243.6	GFP	82.7	jcs	49.8	phenotype	344.9
expressing	241.9	maternal	80.9	Polo	49.8	early	344.8
vector	237.1	Depletion	79.3	orthologue	49.6	show	344.7
cDNA	236.6	T7	77.4	fl	49.3	suggest	344.3
Bristol	235.6	Sohrmann	77.2	loading	49.1	100	343.5

defects	235.2	depletion	76.8	Knockdown	48.4	red	342.7
microscope	231.5	pronuclear	76.0	Simonson	48.4	To	342.7
wild	227.1	nuclei	75.8	NoncommercialSh	47.8	expression	342.6
F1	226.1	knockdown	75.8	S3	47.8	also	342.4
Nomarski	221.4	early	75.5	Dias	47.7	Cell	342.1
stage	218.2	injected	75.4	HTML_ITALICS	47.6	but	341.9
Interference	216.1	RRF	75.2	HTML_ITALICS_g	47.6	both	341.6
transgenic	214.3	231237	74.3	mM	47.2	results	340.6
Center	212.1	interphase	74.3	DAPI	46.5	For	340.4
dpy	211.0	prophase	74.1	Western	46.3	DNA	340.4
unc	210.4	asters	73.2	tubulin	45.6	through	339.9
DAPI	208.7	Kemphues	72.9	knock	45.3	shown	339.8
adults	206.2	arrest	72.5	serum	45.3	controls	339.6
Wild	205.1	condensed	72.2	TIF	45.0	into	339.3
Martinez	203.8	poles	72.0	Goshima	44.9	whether	338.5
rol	202.6	progression	70.8	Jour	44.9	were	338.5
defect	199.4	mitosis	70.2	pgen	44.6	not	338.5
penetrant	199.2	Spindle	69.6	effi	44.6	which	338.5
rrf	197.5	astral	69.5	concanavalin	44.6	can	338.2
depleted	196.4	lapse	69.5	immunoblotting	43.6	These	337.8
arrow	192.4	Kanapin	68.1	Scholey	43.6	has	337.2
fusion	191.7	injection	67.9	siRNA	43.5	7	337.2
promoter	191.4	furrow	67.8	AlikeNo	43.2	PCR	337.1
early	191.1	Strome	67.3	AttributionNoncom	43.2	observed	337.1
wildtype	191.0	spindles	67.3	Unported	43.2	either	336.9
arrest	190.2	doublestranded	67.1	centrosomes	43.2	was	336.1
L1	189.5	stage	66.3	blot	43.1	other	336.1
gfp	189.4	pPD129	66.2	2008	42.9	8	335.9
mediated	189.2	Phenotypes	66.1	Klp10A	42.2	levels	335.5
bacterially	189.0	penetrant	65.9	Healthcare	42.2	at	335.4
n	188.9	progeny	65.8	GTU	42.1	an	335.4
806	186.8	segregation	65.8	codepletion	42.1	C	335.4
thank	186.1	pronucleus	65.7	deplete	42.0	5	335.4
germline	185.6	penetrance	65.6	nity	41.3	or	335.4
arrested	184.8	pole	65.6	Orbit	41.3	3	335.4
allele	182.6	806811	65.6	double	41.3	are	335.4
Dev	182.6	fed	65.1	down	41.2	two	335.3

Our	180.6	granules	65.1	buffer	41.0	green	335.3
depletion	180.0	pronuclei	65.0	unattached	40.9	T7	335.2
penetrance	178.8	metaphase	64.8	anaphase	40.5	using	334.8
Images	178.5	DIC	64.5	Worby	40.5	essential	334.8
Zeiss	178.1	Kohara	64.3	prometaphase	40.5	The	334.6
antibody	177.0	expressing	63.5	Sunkel	40.0	for	334.6
Yuji	175.6	abnormally	63.0	blotting	39.9	from	334.6
failed	175.1	Bot	62.9	Alike	39.3	and	334.6
did	175.1	arrested	62.9	Supplementary	39.0	4	334.6
fluorescence	174.6	rrf	62.8	tagged	38.0	of	334.6
microscopy	173.9	Curr	62.5	PBS	37.9	to	334.6
854	173.0	defect	62.4	depolymerize	37.8	A	334.6
103112	172.7	Xu	61.4	S1	37.6	be	334.6
prophase	172.3	daughter	61.4	Materials	37.4	a	334.6
young	170.5	meiosis	61.4	g	37.3	in	334.6
examined	166.8	stages	60.2	treatments	37.3	on	334.6
arrowhead	165.9	Consistent	60.0	templates	36.9	is	334.6
clones	165.4	Hyman	59.8	specifi	36.8	1	334.6
Strome	163.9	T3	58.2	Sigma	36.8	2	334.6
806811	163.8	Parrish	57.9	uence	36.5	by	334.6
Bowerman	163.7	Tijsterman	57.7	affi	36.5	that	334.6
larvae	162.6	Simmer	57.5	Rieder	36.4	as	334.6
mitotic	162.5	pL4440	57.3	midbody	36.3	the	334.6
811	161.3	nuclear	57.0	objective	36.2	with	334.6
stages	160.5	redundantly	56.9	Stuurman	36.2	analysis	333.8
T3	160.1	Rybina	56.7	Buster	36.2	localization	333.7
divisions	160.0	13171319	56.7	Wordeman	36.2	between	333.6
Sohrmann	160.0	Dev	56.6	Dixon	36.2	these	333.5
division	159.5	centrosomes	56.3	Tubulin	35.9	data	333.4
reporter	159.3	antibody	56.1	amplifi	35.8	this	333.3
plates	159.1	Embryonic	56.0	CELL	35.7	formation	332.6
anaphase	159.0	strains	56.0	g005	35.6	In	332.5
fed	158.8	depleted	55.4	fi	35.6	independent	332.3
pRF4	155.4	diakinesis	55.1	ZW10	35.6	D	332.2
null	154.6	Mitotic	55.1	g003	35.4	This	332.0
pk1426	153.1	806	54.1	g002	35.4	Dev	331.8
ed3	153.1	fission	54.1	dissect	35.3	20	331.0

maternal	151.5	F1		53.8	lysates		35.1	our		330.4
Seydoux	151.2	essential		53.8	Plan		35.1	have		329.7
amplified	150.1		811	53.6	medium		35.0		6	329.7
marker	150.0	Loss		53.4	Images		34.9	mechanism		329.5
nuclear	149.3	Systematic		53.4	colchicine		34.8		10	328.9
germ	149.2	rol		53.3	g004		34.7	its		328.5
Poulin	148.1	germ		53.2	Mirror		34.7	Materials		328.1
sterility	147.9	DE3		53.1		2009	34.7	all		327.6
late	147.0	Dong		52.9	mammalian		34.3	been		327.1
Discussion	145.8	Centrosome		52.8	fetal		34.3	type		327.0
Genetics	145.7	Kirkham		52.7	defi		34.2	B		326.6
essential	145.6	N2		52.7	washed		34.2	containing		326.5
pie	145.1	Seydoux		52.6	Carl		34.1		12	325.7
DE3	144.9	microscopy		52.1	subunits		34.1	it		324.5
encodes	144.4	Yuji		51.5	sample		34.1	than		324.5
Double	144.2	zygotic		51.2	g001		34.0	effect		324.5
knockdown	143.8	cyclin		51.2	quantifi		34.0		30	323.7
Depletion	143.3	microtubules		51.1	stably		34.0	normal		322.9
meiotic	142.8	seam		51.1	JOURNAL		34.0	lethality		322.6
metaphase	142.8	resulted		51.1	journal		33.5	associated		322.5
antibodies	142.7	wild		51.1	V5		33.5	reduction		321.3
adult	141.5	PAR		50.9	spinning		33.4	their		320.5
gift	140.7	onecell		50.9	GE		33.3	E		320.0
performed	140.4	vulva		50.9	kinesins		32.7	Invitrogen		319.6
larval	139.5	failed		50.8	Aurora		32.4	addition		319.2
Consistent	138.5	MEL		50.6	eld		32.4	when		319.1
Collar	138.2	oocytes		50.5	synthesized		32.4	each		318.9
primers	138.1	zygote		50.3	precipitated		32.4	found		318.5
panels	137.6	nucleus		50.2	orthologues		32.3	consistent		318.0
arrows	137.6	staining		50.1	incubation		32.1	only		318.0
optics	135.9	RNAitreated		49.9	Submitted		32.1	incubated		317.7
treated	135.8	Nomarski		49.9	CENP		32.1	images		317.3
anti	134.9	chromatids		49.5	Khodjakov		32.1	culture		317.3
Simmer	134.4	Srayko		49.5	license		32.1	directly		317.0
plasmid	134.1	localization		49.2	Claudio		32.1	embryo		316.7
transgene	133.5	Amores		49.1	monopolar		32.0	targeting		316.7
camera	133.2	prometaphase		49.1	SL2		32.0	known		316.5

extrachromosoma	132.8	ampicillin	48.9	untransfected	32.0	regulates		316.4
granules	132.5	sterile	48.7	beads	32.0		14	315.7
cloned	131.5	Wild	48.5	throughput	31.9	expressed		315.6
Gotta	131.5	oocyte	48.1	nc	31.8	against		315.4
localized	131.0	Defects	47.6	Earnshaw	31.8		13	314.6
Development	131.0	tubulin	47.6	Treatment	31.7	then		314.0
Genes	129.7	timing	47.4	Gibco	31.7	microscope		313.5
animals	129.3	deconvolved	47.4	permeabilized	31.7	three		313.0
normal	129.3	furrows	47.4	quantification	31.5	F		313.0
severe	128.0	protruding	47.0	signifi	31.2	suggesting		312.4
Accepted	127.4	Embryo	47.0	Ctrl	31.0	well		311.9
fused	127.3	eggs	47.0	PAGE	30.8	more		311.4
mutant	127.3	Moreno	46.9	Bousbaa	30.8	further		311.3
lethal	126.6	meiotic	46.8	pAc	30.8	see		311.0
image	126.0	spermatheca	46.7	Abcam	30.8	indicated		310.8
Resources	125.9	adults	46.6	antibodies	30.7	embryonic		310.7
abnormal	125.2	germline	46.6	checkpoint	30.7	indicating		310.6
loss	123.9	roles	46.6	Trizol	30.7	As		310.6
Kemphues	123.9	proper	46.3	Salmon	30.7	resulted		308.9
ampicillin	123.9	images	46.3	g006	30.7	However		308.7
localization	123.6	annealed	46.2	bovine	30.3	indicate		307.5
controls	123.5	condensation	46.2	unfocused	30.1	G		307.4
doublestranded	122.8	fusion	46.0	classifi	30.1	M		307.2
RESEARCH0002	122.7	kinetochore	45.9	Hoyt	30.1	Our		307.1
vulva	122.5	Koushika	45.7	Logarinho	30.0		15	306.9
reduced	122.5	deplete	45.7	Ran	30.0	transcription		306.5
spindle	122.4	Schnabel	45.6	Mitchison	30.0	compared		306.4
morphogenesis	122.2	Hird	45.4	lysis	29.9		11	306.3
cytokinesis	121.4	chromatin	45.2	immunofl	29.9	contrast		306.1
resulted	121.3	reporter	45.1	Kc	29.8	similar		305.9
DC1	121.2	unc	45.0	Oegema	29.7	caused		305.5
1974	120.5	Rothman	44.8	Cytokinesis	29.7	functional		305.2
Parrish	120.2	somatic	44.7	knocking	29.6	vivo		304.4
reduction	120.2	pseudocleavage	44.6	congression	29.6	showed		304.3
ml	119.8	cki	44.6	SDS	29.5	cDNA		303.8
seam	119.7	mothers	44.4	Verhagen	29.3	target		303.8
RRF	119.7	brood	44.1	Arf1	29.3	Sigma		303.7

Microscopy		119.6	larvae		44.0	KLP10A		29.3	detected		303.6
conserved		119.4	Encalada		43.7	Dzhindzhev		29.3	signal		303.4
Results		119.2	Gnczy		43.7	okadaic		29.3	human		303.4
construct		118.8	abnormal		43.4	jcb		29.1		25	302.9
conclude		118.5	centrosome		43.3	instructions		28.9	involved		302.6
lines		118.2	gfp		43.3	qRT		28.9	following		302.6
Curr		118.1	larval		43.2	chromatid		28.9		9	302.2
Priess		117.4	dpy		42.6	tension		28.7	specific		302.2
disorganized		117.1	redundant		42.6	Aldrich		28.7	wild		301.9
background		116.6	Double		42.5	normalized		28.3	components		301.8
Loss		116.2	microscope		42.4	Use		28.3		16	301.6
appeared		116.1	failure		42.4	Click		28.3	requires		301.4
	395	116.0	syncytial		42.4	grown		28.3	microscopy		300.7
DNA		115.9	maternally		42.2	VOLUME		28.0	P		300.7
Ambion		115.7	aster		42.1	Triton		27.9	down		300.6
fragment		115.6	Hamill		41.9	spindle		27.8	transfected		299.6
Gonads		115.5		421	41.9	S4		27.8	progression		299.2
Phenotypes		114.4	laid		41.6	extracts		27.6	activation		299.0
zygotic		113.6	pk1426		41.4	ndings		27.5	Curr		299.0
adulthood		113.2	telophase		41.4	rmed		27.5	mutants		298.0
spermatheca		112.6	regulate		41.4	Kc167		27.5	any		297.5
Casey		112.6		408	41.4	http		27.5	g		297.4
mothers		112.0	viability		41.3	After		27.4	loss		296.7
annealed		111.9	Gonczy		41.3	Biosystems		27.2	respectively		296.6
hypodermis		111.9	qC1		41.1	Habermann		27.2	S		296.3
20C		111.8	immunofluorescen		41.0	MAST		27.2	important		296.2
transferred		111.8	stained		41.0	ml		27.1	together		295.3
immunostaining		111.7	component		40.9	silencing		27.0	domain		294.7
green		111.5	silencing		40.6	wells		27.0	mutant		294.7
Arrows		111.3	fused		40.6		1371	27.0	mechanisms		294.4
pronuclei		111.0	late		40.6	progression		27.0	complexes		294.1
contrast		111.0	Asymmetric		40.6	conserved		26.8	blue		293.9
polymerase		110.8	arrowheads		40.4	harvested		26.7	interaction		293.6
Animals		110.6	restrictive		40.2		1993	26.6	genes		293.5
National		110.5	checkpoint		40.1	Antibodies		26.5	process		293.1
S1		110.3	initiates		40.1	file		26.4	Ambion		293.1
regulates		110.2		325330	40.1	Interference		26.4	mitotic		293.0

mitosis	110.0	Polarity	40.1	depleting	26.3	melanogaster	292.7
Kimble	109.9	scored	40.1	xed	26.3	DAPI	292.2
pronuclear	109.8	missegregation	40.1	centromeres	26.1	supported	291.1
Spindle	109.8	envelope	40.1	spindles	26.0	presence	290.9
Adobe	109.7	Carter	40.1	CuSO	25.9	such	290.0
Praitis	109.3	delay	40.0	JCB	25.9	regulated	289.9
pPD95	109.1	specification	40.0	Rod	25.9	Consistent	289.8
meiosis	109.0	normal	39.4	bipolarity	25.9	Genes	289.8
proper	108.7	Oegema	39.3	THE	25.9	additional	289.6
4B	108.5	Kipreos	39.2	transfections	25.9	absence	289.4
nucleus	107.9	Nasmyth	39.2	Transfected	25.9	Cells	289.1
CC	107.9	nocodazole	39.2	subunit	25.8	Introduction	288.3
Oegema	107.5	Geraldine	39.2	inhibitor	25.8	cultured	288.3
homozygotes	107.4	5E	39.0	S6	25.6	may	287.9
supported	107.2	postembryonic	39.0	DRSC	25.6	After	287.7
homolog	107.1	hypersensitive	38.9	Deltavision	25.6	no	287.3
normally	106.9	localizes	38.9	inhibitors	25.5	distinct	287.3
arm	106.8	Required	38.7	confi	25.5	50	286.8
blastomere	106.8	depleting	38.7	NUMBER	25.5	component	286.6
worms	106.5	Schetter	38.7	cient	25.4	level	286.5
segregation	106.4	Aurora	38.7	suffi	25.4	same	286.5
somatic	106.4	chromatid	38.7	BubR1	25.4	one	286.5
Axioplan	106.2	927946	38.7	anaphases	25.4	whereas	285.8
Photoshop	106.1	AZ212	38.7	deconvolved	25.4	region	285.5
poles	106.0	morphogenesis	38.6	siRNAs	25.2	examined	285.3
homozygous	105.6	Center	38.5	disassembly	25.1	tubulin	285.2
redundantly	105.6	LET	38.4	protease	25.0	different	284.8
su1006	105.5	Tabara	38.4	1083	25.0	H	284.7
M9	105.5	regulators	38.3	validate	24.9	panel	284.2
interacts	105.4	regresses	38.1	nM	24.8	blot	284.2
Embryonic	105.3	furrowing	38.1	ds	24.8	time	283.9
hatching	105.3	fertilized	38.1	incubated	24.7	panels	283.8
laid	105.1	DM1A	38.1	WSD	24.5	induced	283.2
lapse	105.0	RESEARCH0002	38.1	WSD_gene_dmela	24.5	like	282.5
Hardin	105.0	elt	37.7	SYBR	24.3	generated	282.3
hypodermal	104.7	Kimble	37.7	TE2000	24.2	I	282.2
immunofluorescer	104.5	regressed	37.6	Zwilch	24.2	ml	282.2

oocyte		104.3	cleavage		37.6	polymerizing		24.2	studies		282.0
mounted		103.9	Development		37.6	fmk		24.2	does		281.6
	12171226	103.7		395	37.5	Yokogawa		24.2	N		280.5
Brenner		103.3	Gene		37.4	Culture		24.2	et		280.4
hT2		103.3	requirement		37.3	PFA		24.2	R		280.3
domain		103.2	Bristol		37.3	Introduction		24.1	vector		278.9
bars		103.2	ruIs32		37.2	centralspindlin		24.1	development		278.1
transformed		102.8	Kaletta		37.2	DEVDase		24.1	lines		278.1
exon		102.8	microtubule		36.9	Mx3000P		24.1	factor		278.1
incubated		102.4	promoters		36.7	prostaglandin		24.1	L		277.7
	0	102.1	null		36.7	ECT2		24.1	could		277.2
We		101.9	uninjected		36.5	polewards		24.1	al		277.0
focal		101.8	completion		36.3	overcondensation		24.1	vitro		276.7
affected		101.7	plates		36.2	depolymerase		24.1	Y		276.3
rabbit		101.4	CUL		36.2	pAC		24.1	determine		276.1
4A		101.1	cul		36.2		3790	24.1	before		275.9
RT		101.1	ZYG		36.2	Steuer		24.1	indicates		275.6
4C		100.9	dynactin		36.2	codepleted		24.1	targets		275.2
LGIII		100.8	Knockout		36.2	bioconductor		24.1	fixed		275.2
Austin		100.3	localize		36.1	widefield		24.1	total		274.2
Creation		100.3	Injection		36.1	TAATACGACTCA		24.1	work		274.0
regulator		100.3	redundancy		36.0	cytokinesis		24.0	suggests		273.4
NIH		100.2	Slides		36.0	S5		24.0	regulators		273.3
3A		100.2	Grishok		36.0	Saitoh		24.0	yeast		272.6
progression		100.0	Greenstein		36.0	Cycle		23.9	identified		272.3
envelope		100.0	homozygous		35.9	seeded		23.9		24	272.2
silencing		99.9	mediated		35.7	Stable		23.9	system		272.1
upstream		99.6	Abnormal		35.7	Spindle		23.9	mouse		271.9
rescued		99.6	pha		35.7	material		23.9	line		270.5
postembryonic		99.5	microinjected		35.2	added		23.8		21	270.2
Required		99.4	functions		35.1	Genet		23.8	All		269.9
cosmid		99.4	transition		35.0	coated		23.8	they		269.7
suggest		98.9	budding		34.9	Roche		23.6	Western		269.3
bar		98.5	Cytokinesis		34.9	triplicate		23.5	increased		268.6
bacteria		98.4	zyg		34.9	Hergert		23.5	had		268.3
rescue		97.8	Rougvie		34.8	Moutinho		23.5	failed		267.2
defective		97.7	bacteria		34.8	Gorbsky		23.5	targeted		266.9

type	97.6	implicated	34.5	CLASP	23.5	primers	266.8
control	97.6	Knockdown	34.4	chromatinassociat	23.5	rabbit	266.3
Dong	97.4	reduction	34.4	Immunoelectron	23.5	Interestingly	266.2
5A	96.9	spd	34.4	CellTiter	23.5	22	265.6
e189	96.5	cDNA	34.3	Jafrac2	23.5	corresponding	265.5
7194	96.3	Lyczak	34.2	cohesins	23.5	nuclei	264.4
suppressed	96.2	Kirchner	34.2	unidentifi	23.5	result	263.9
Control	96.2	Willis	34.2	rnai	23.5	T	263.8
methanol	96.2	Glotzer	34.2	nonspecifi	23.5	affect	263.8
functions	96.0	normally	34.2	Sarstedt	23.5	mM	263.7
kb	96.0	onset	34.2	Resource	23.5	stages	263.2
S2	95.9	PGL	34.1	York	23.4	study	263.2
caused	95.6	Immunofluorescen	34.1	anterior	23.3	secondary	262.9
predicted	95.5	Quintin	34.0	primers	23.3	PBS	262.7
indicated	94.8	PIE	34.0	uorescence	23.3	including	262.7
To	94.3	templates	34.0	ciency	23.3	out	262.4
posterior	94.3	posterior	33.9	Kinetochore	23.3	buffer	261.5
visible	94.3	L1	33.9	Basto	23.3	K	261.0
4D	94.3	hatch	33.7	multipolar	23.3	multiple	260.6
required	94.3	green	33.7	Sampaio	23.3	obtained	259.5
Immunostaining	94.2	Microscopy	33.6	6P	23.3	regulating	259.5
Kanapin	94.1	regulates	33.6	Knock	23.3	novel	259.4
MEGAscript	94.0	Mango	33.6	purifi	23.3	X	259.3
PGL	93.9	adult	33.6	Kapoor	23.3	J	259.2
promotes	93.7	transformed	33.5	Meier	23.3	fusion	258.9
421	93.7	severe	33.3	REPORT	23.2	might	258.6
backgrounds	93.6	CC	33.3	poleward	23.2	some	258.1
Schnabel	93.6	targeted	33.1	Interphase	23.2	2005	258.0
anterior	93.5	vector	33.0	immunofluorescen	23.1	2004	257.6
Immunofluorescer	93.1	Schumacher	32.9	microscope	23.1	times	257.4
carrying	92.8	cyk	32.8	Applied	23.0	implicated	257.1
Nature	92.7	Maddox	32.8	metaphase	23.0	where	256.6
L3	92.6	depletions	32.8	microscopy	22.9	extracts	256.5
redundant	92.6	potent	32.8	complexes	22.8	Mol	255.7
maintenance	92.5	Iain	32.8	poles	22.8	stage	254.8
Hyman	92.5	breakdown	32.8	co	22.7	present	253.6
genetic	92.4	pRF4	32.6	cant	22.6	several	253.6

Bot	92.2	genetic	32.6	software	22.3	second	252.3
3B	91.8	reduced	32.6	1992	22.3	possibility	251.4
fixed	91.7	prevents	32.6	centrifugation	22.2	upstream	251.1
manuscript	91.6	optics	32.5	2006	22.1	http	251.0
Embryo	91.6	upstream	32.2	rst	22.1	left	250.7
Rothman	91.4	hatching	32.1	inhibition	22.1	sequence	250.7
suggesting	91.1	Improvision	32.1	pp	22.1	regulator	250.3
Scale	91.1	Microtubules	32.1	Tenev	22.1	revealed	250.1
cytoplasm	91.1	severing	32.1	RNAitreated	22.1	right	249.7
histone	90.8	Pvl	32.0	associates	22.1	events	249.1
cells	90.8	Metaphase	32.0	EB1	22.0	alone	249.0
Antibodies	90.7	fertile	31.9	vitro	21.9	17	248.3
EMBO	90.4	cosmid	31.9	DOI	21.9	Center	248.0
cgi	90.3	adulthood	31.8	cultures	21.9	upon	248.0
abnormally	90.0	Ambion	31.8	Rep	21.9	promoter	248.0
oocytes	89.8	Sunkel	31.7	Glover	21.8	Images	247.9
Genotype	89.7	ingressed	31.7	HeLa	21.8	cytoplasmic	247.7
Sugimoto	89.7	syntaxins	31.7	panels	21.7	s	247.2
clone	89.6	ISREC	31.7	experiments	21.7	kinase	247.1
later	89.4	pPD128	31.7	panel	21.6	mRNA	247.0
Tijsterman	89.2	4525	31.7	Gomes	21.5	direct	246.9
regulate	89.0	antibodies	31.7	emboj	21.5	pathways	246.9
MK	88.9	cdc2	31.6	H3	21.4	number	246.6
glp	88.9	YP170	31.6	1994	21.3	genetic	246.5
binding	88.9	NEBD	31.6	Cid	21.2	Thus	245.4
Injection	88.8	12091226	31.6	cation	21.2	single	245.4
eggs	88.8	cye	31.6	fibroblasts	21.2	relative	245.3
Mol	88.6	separase	31.6	qPCR	21.2	recruitment	243.9
integrated	88.5	examined	31.5	S7	21.1	factors	243.8
Injected	88.5	soaking	31.4	camera	21.1	within	243.2
indicate	88.3	gonads	31.3	Experimental	21.0	first	243.0
experiments	88.3	asymmetrically	31.2	Calbiochem	21.0	mitosis	243.0
hatched	87.7	arrowhead	31.2	depletions	21.0	evidence	242.6
compared	87.5	encodes	31.1	quantitative	20.7	negative	242.5
genomic	87.1	Nature	31.1	attachment	20.7	interact	241.9
telophase	87.1	dpy10	31.0	untagged	20.7	possible	241.1
cytoplasmic	87.0	Hagstrom	31.0	apoptotic	20.6	least	240.9

confocal	86.7	Arshad	31.0	g007		20.5	larvae	240.9
dissected	86.2	Tenenhaus	31.0		1999	20.4	assembly	240.6
mutants	86.1	worms	31.0	fraction		20.4	form	240.5
