# Supplementary material for: Automatic categorization of diverse experimental information in the bioscience literature
Source: BMC Bioinformatics. 2012 Jan 26;13:16. doi: 10.1186/1471-2105-13-16 (PMC3305665; doi:10.1186/1471-2105-13-16)
Supplement: Additional file 14 — easySVM.tar.gz. This file contains instructions, code and samples set for text categorization described here. Please read the file named INSTRUCTION under the directory easySVM after decompressing the tar ball before running the analyses. [file 1471-2105-13-16-S14.ZIP › easySVM-1.tar!/easySVM/make_training_vectors/bp/training_indices/HTML]

WBPaper00028343,1,3170
WBPaper00028792,4,2871
WBPaper00028366,3,8139
