# Supplementary material for: Automatic categorization of diverse experimental information in the bioscience literature
Source: BMC Bioinformatics. 2012 Jan 26;13:16. doi: 10.1186/1471-2105-13-16 (PMC3305665; doi:10.1186/1471-2105-13-16)
Supplement: Additional file 14 — easySVM.tar.gz. This file contains instructions, code and samples set for text categorization described here. Please read the file named INSTRUCTION under the directory easySVM after decompressing the tar ball before running the analyses. [file 1471-2105-13-16-S14.ZIP › easySVM-1.tar!/easySVM/make_training_vectors/bp/training_indices/html]

WBPaper00031442,1,8179
WBPaper00029059,1,12736
WBPaper00028811,1,7260
WBPaper00030985,1,11395
WBPaper00029344,1,11324
WBPaper00031597,1,7482
WBPaper00028994,2,14527
WBPaper00031478,1,13073
WBPaper00028854,1,11619
WBPaper00031319,1,10996
WBPaper00031009,1,3518
WBPaper00031449,1,9349
WBPaper00029190,2,9156
WBPaper00029396,1,14251
WBPaper00031316,1,10007
WBPaper00028792,1,2871
WBPaper00031485,1,13034
WBPaper00028366,1,8139
WBPaper00031204,3,6871
WBPaper00030781,1,15146
WBPaper00030909,3,13434
WBPaper00029148,1,9695
WBPaper00029234,1,11171
WBPaper00028771,1,7614
WBPaper00030923,2,11924
WBPaper00031436,2,14077
WBPaper00028816,1,5924
