# Supplementary material for: Automatic categorization of diverse experimental information in the bioscience literature
Source: BMC Bioinformatics. 2012 Jan 26;13:16. doi: 10.1186/1471-2105-13-16 (PMC3305665; doi:10.1186/1471-2105-13-16)
Supplement: Additional file 14 — easySVM.tar.gz. This file contains instructions, code and samples set for text categorization described here. Please read the file named INSTRUCTION under the directory easySVM after decompressing the tar ball before running the analyses. [file 1471-2105-13-16-S14.ZIP › easySVM-1.tar!/easySVM/analyze_new_papers/FAQ.html]

LIBSVM FAQ

 **# LIBSVM FAQ** 
**last modified :** 
Thu, 19 Mar 2009 12:30:41 GMT
- All Questions(72)

**- Q1:\_Some\_sample\_uses\_of\_libsvm(2)
- Q2:\_Installation\_and\_running\_the\_program(9)
- Q3:\_Data\_preparation(6)
- Q4:\_Training\_and\_prediction(33)
- Q5:\_Probability\_outputs(3)
- Q6:\_Graphic\_interface(3)
- Q7:\_Java\_version\_of\_libsvm(4)
- Q8:\_Python\_interface(5)
- Q9:\_MATLAB\_interface(7)** 


- Some courses which have used libsvm as a tool
- Some applications which have used libsvm
- Where can I find documents of libsvm ?
- Where are change log and earlier versions?
- I would like to cite LIBSVM. Which paper should I cite?
- I would like to use libsvm in my software. Is there any license problem?
- Is there a repository of additional tools based on libsvm?
- On unix machines, I got "error in loading shared libraries" or "cannot open shared object file." What happened ?
- I have modified the source and would like to build the graphic interface "svm-toy" on MS windows. How should I do it ?
- I am an MS windows user but why only one (svm-toy) of those precompiled .exe actually runs ?
- What is the difference between "." and "\*" outputed during training?
- Why sometimes not all attributes of a data appear in the training/model files ?
- What if my data are non-numerical ?
- Why do you consider sparse format ? Will the training of dense data be much slower ?
- Why sometimes the last line of my data is not read by svm-train?
- Is there a program to check if my data are in the correct format?
- May I put comments in data files?
- The output of training C-SVM is like the following. What do they mean?
- Can you explain more about the model file?
- Should I use float or double to store numbers in the cache ?
- How do I choose the kernel?
- Does libsvm have special treatments for linear SVM?
- The number of free support vectors is large. What should I do?
- Should I scale training and testing data in a similar way?
- Does it make a big difference if I scale each attribute to [0,1] instead of [-1,1]?
- The prediction rate is low. How could I improve it?
- My data are unbalanced. Could libsvm handle such problems?
- What is the difference between nu-SVC and C-SVC?
- The program keeps running (without showing any output). What should I do?
- The program keeps running (with output, i.e. many dots). What should I do?
- The training time is too long. What should I do?
- Does shrinking always help?
- How do I get the decision value(s)?
- How do I get the distance between a point and the hyperplane?
- On 32-bit machines, if I use a large cache (i.e. large -m) on a linux machine, why sometimes I get "segmentation fault ?"
- How do I disable screen output of svm-train and svm-predict ?
- I would like to use my own kernel but find out that there are two subroutines for kernel evaluations: k\_function() and kernel\_function(). Which one should I modify ?
- What method does libsvm use for multi-class SVM ? Why don't you use the "1-against-the rest" method ?
- After doing cross validation, why there is no model file outputted ?
- Why my cross-validation results are different from those in the Practical Guide?
- But on some systems CV accuracy is the same in several runs. How could I use different data partitions?
- I would like to solve L2-loss SVM (i.e., error term is quadratic). How should I modify the code ?
- How do I choose parameters for one-class svm as training data are in only one class?
- Why the code gives NaN (not a number) results?
- Why on windows sometimes grid.py fails?
- Why grid.py/easy.py sometimes generates the following warning message?
- Why the sign of predicted labels and decision values are sometimes reversed?
- I don't know class labels of test data. What should I put in the first column of the test file?
- How can I use OpenMP to parallelize LIBSVM on a multicore/shared-memory computer?
- How could I know which training instances are support vectors?
- Why training a probability model (i.e., -b 1) takes a longer time?
- Why using the -b option does not give me better accuracy?
- Why using svm-predict -b 0 and -b 1 gives different accuracy values?
- How can I save images drawn by svm-toy?
- I press the "load" button to load data points but why svm-toy does not draw them ?
- I would like svm-toy to handle more than three classes of data, what should I do ?
- What is the difference between Java version and C++ version of libsvm?
- Is the Java version significantly slower than the C++ version?
- While training I get the following error message: java.lang.OutOfMemoryError. What is wrong?
- Why you have the main source file svm.m4 and then transform it to svm.java?
- On MS windows, why does python fail to load the pyd file?
- How to modify the python interface on MS windows and rebuild the .pyd file ?
- Except the python-C++ interface provided, could I use Jython to call libsvm ?
- How could I install the python interface on Mac OS?
- I typed "make" on a unix system, but it says "Python.h: No such file or directory?"
- I compile the MATLAB interface without problem, but why errors occur while running it?
- On 64bit Windows I compile the MATLAB interface without problem, but why errors occur while running it?
- Does the MATLAB interface provide a function to do scaling?
- How could I use MATLAB interface for parameter selection?
- I use MATLAB parallel programming toolbox on a multi-core environment for parameter selection. Why the program is even slower?
- How could I generate the primal variable w of linear SVM?
- Is there an OCTAVE interface for libsvm?

---


**Q: Some courses which have used libsvm as a tool**
  

- Institute for Computer Science,
  Faculty of Applied Science, University of Freiburg, Germany
  - Division of Mathematics and Computer Science.
    Faculteit der Exacte Wetenschappen
    Vrije Universiteit, The Netherlands. - Electrical and Computer Engineering Department,
      University of Wisconsin-Madison
      - Technion (Israel Institute of Technology), Israel.- Computer and Information Sciences Dept., University of Florida- The Institute of Computer Science,
            University of Nairobi, Kenya.- Applied Mathematics and Computer Science, University of Iceland.- SVM tutorial in machine learning
                summer school, University of Chicago, 2005.

[Go Top]


---


**Q: Some applications which have used libsvm** 
  

- Realtime object recognition

[Go Top]


---


**Q: Where can I find documents of libsvm ?**
  

In the package there is a README file which
details all options, data format, and library calls.
The model selection tool and the python interface
have a separate README under the directory python.
The guide
A practical guide to support vector classification
 shows beginners how to train/test their data.
The paper LIBSVM
: a library for support vector machines discusses the implementation of
libsvm in detail.

[Go Top]


---


**Q: Where are change log and earlier versions?**
  

See the change log.

You can download earlier versions
here.

[Go Top]


---


**Q: I would like to cite LIBSVM. Which paper should I cite?** 
  

Please cite the following document:

Chih-Chung Chang and Chih-Jen Lin, LIBSVM
: a library for support vector machines, 2001.
Software available at http://www.csie.ntu.edu.tw/~cjlin/libsvm

The bibtex format is

```
@Manual{CC01a,
  author =	 {Chih-Chung Chang and Chih-Jen Lin},
  title =	 {{LIBSVM}: a library for support vector machines},
  year =	 {2001},
  note =	 {Software available at \url{http://www.csie.ntu.edu.tw/~cjlin/libsvm}}
}
```

[Go Top]


---


**Q: I would like to use libsvm in my software. Is there any license problem?**
  

The libsvm license ("the modified BSD license")
is compatible with many
free software licenses such as GPL. Hence, it is very easy to
use libsvm in your software.
It can also be used in commercial products.

[Go Top]


---


**Q: Is there a repository of additional tools based on libsvm?**
  

Yes, see libsvm
tools

[Go Top]


---


**Q: On unix machines, I got "error in loading shared libraries" or "cannot open shared object file." What happened ?** 
  

This usually happens if you compile the code
on one machine and run it on another which has incompatible
libraries.
Try to recompile the program on that machine or use static linking.

[Go Top]


---


**Q: I have modified the source and would like to build the graphic interface "svm-toy" on MS windows. How should I do it ?**
  

Build it as a project by choosing "Win32 Project."
On the other hand, for "svm-train" and "svm-predict"
you want to choose "Win32 Console Project."
After libsvm 2.5, you can also use the file Makefile.win.
See details in README.

If you are not using Makefile.win and see the following
link error

```
LIBCMTD.lib(wwincrt0.obj) : error LNK2001: unresolved external symbol
_wWinMain@16
```

you may have selected a wrong project type.

[Go Top]


---


**Q: I am an MS windows user but why only one (svm-toy) of those precompiled .exe actually runs ?** 
  

You need to open a command window
and type svmtrain.exe to see all options.
Some examples are in README file.

[Go Top]


---


**Q: What is the difference between "." and "\*" outputed during training?** 
  

"." means every 1,000 iterations (or every #data
iterations is your #data is less than 1,000).
"\*" means that after iterations of using
a smaller shrunk problem,
we reset to use the whole set. See the
implementation document for details.

[Go Top]


---


**Q: Why sometimes not all attributes of a data appear in the training/model files ?**
  

libsvm uses the so called "sparse" format where zero
values do not need to be stored. Hence a data with attributes

```
1 0 2 0
```

is represented as

```
1:1 3:2
```

[Go Top]


---


**Q: What if my data are non-numerical ?**
  

Currently libsvm supports only numerical data.
You may have to change non-numerical data to
numerical. For example, you can use several
binary attributes to represent a categorical
attribute.

[Go Top]


---


**Q: Why do you consider sparse format ? Will the training of dense data be much slower ?**
  

This is a controversial issue. The kernel
evaluation (i.e. inner product) of sparse vectors is slower
so the total training time can be at least twice or three times
of that using the dense format.
However, we cannot support only dense format as then we CANNOT
handle extremely sparse cases. Simplicity of the code is another
concern. Right now we decide to support
the sparse format only.

[Go Top]


---


**Q: Why sometimes the last line of my data is not read by svm-train?**
  

We assume that you have '\n' in the end of
each line. So please press enter in the end
of your last line.

[Go Top]


---


**Q: Is there a program to check if my data are in the correct format?**
  

The svm-train program in libsvm conducts only a simple check of the input data. To do a
detailed check, after libsvm 2.85, you can use the python script tools/checkdata.py. See tools/README for details.

[Go Top]


---


**Q: May I put comments in data files?**
  

No, for simplicity we don't support that.
However, you can easily preprocess your data before
using libsvm. For example,
if you have the following data

```
test.txt
1 1:2 2:1 # proten A
```

then on unix machines you can do

```
cut -d '#' -f 1 < test.txt > test.features
cut -d '#' -f 2 < test.txt > test.comments
svm-predict test.feature train.model test.predicts
paste -d '#' test.predicts test.comments | sed 's/#/ #/' > test.results
```

[Go Top]


---


**Q: The output of training C-SVM is like the following. What do they mean?**
  
  
optimization finished, #iter = 219
  
nu = 0.431030
  
obj = -100.877286, rho = 0.424632
  
nSV = 132, nBSV = 107
  
Total nSV = 132

obj is the optimal objective value of the dual SVM problem.
rho is the bias term in the decision function
sgn(w^Tx - rho).
nSV and nBSV are number of support vectors and bounded support
vectors (i.e., alpha\_i = C). nu-svm is a somewhat equivalent
form of C-SVM where C is replaced by nu. nu simply shows the
corresponding parameter. More details are in
libsvm document.

[Go Top]


---


**Q: Can you explain more about the model file?**
